# Nebraska

**Published:** 1896-06

**Authors:** 


*Nebraska.* The Nebraska Experiment Station for the past year has been working on the lines of an antitoxin for hog-cholera, and so far with very gratifying results. Pregnant sows and some hogs affected at the time of treatment have been experimented with, in all of which promising favorable results were obtained.

